# High-throughput DNA sequencing of microbiota at interproximal sites

**DOI:** 10.1080/20002297.2019.1687397

**Published:** 2019-11-11

**Authors:** Miguel Carda-Diéguez, Luis Alberto Bravo-González, Isabel María Morata, Ascensión Vicente, Alex Mira

**Affiliations:** a Genomics & Health Department, FISABIO Institute, Valencia, Spain; b Department of Orthodontics, Faculty of Medicine, University of Murcia, Murcia, Spain

**Keywords:** Caries, 16S rRNA, next generation sequencing, interproximal, microbiota

## Abstract

**Objective:** The oral microbiota has been deeply studied by high-throughput sequencing techniques. However, although the interproximal regions have one of the highest caries rates in the oral cavity, information about the bacterial composition at those sites is scarce.

**Methods:** In this study, we used 16S rRNA Illumina sequencing to describe the microbiota associated to interproximal regions at two time points. In addition, dental plaque samples at the vestibular and lingual surfaces from the same teeth were also analysed at the two time points.

**Results:** Interproximal-associated microbiota was found to be similar to already described bacterial communities in other mouth niches. *Streptoccocus, Veillonella, Rothia, Actinomyces, Neisseria, Haemophilus* and *Fusobacterium* were the most abundant genera in this oral region. Statistical analyses showed that the microbiota from interproximal sites was more similar to that sampled from the vestibular surfaces than to the lingual surfaces. Interestingly, many potentially cariogenic bacteria such as *Scardovia*, *Atopobium* or *Selenomonas* were over-represented in the interproximal regions in comparison with vestibular and lingual sites.

**Conclusion:** The microbiota at interproximal regions appears to be specific and stable through time. Potentially pathogenic bacteria may increase caries development risk and gingival inflammation at those sites.

## Introduction

During the last decade, important efforts have been made to understand the composition and ecology of the microbiota in the oral cavity [1,2]. Although the oral microbiome is considered one of the most stable human-associated microbiotas, the variability of ecological conditions that can be found in the different regions in the mouth makes the oral microbiota diverse and niche-specific [3,4]. For example, although the vestibular and lingual regions of teeth are relatively close and one could expect them to share to a certain extent their microbial profile, the lingual contact seemed to strongly affect the *Streptococcus* and *Fusobacterium* abundance, among other microorganisms [5]. This variability among oral niches has increased the difficulty to study the oral microbiota, and a more precise, site-specific sampling has been proposed [6].

It is well established that the microbial composition in the oral cavity has severe health implications both locally and systemically [7]. In fact, caries and periodontitis are among the most prevalent chronic diseases worldwide (https://www.who.int/oral_health/disease_burden/global/en/) and both develop as a consequence of bacterial metabolism [3,8]. Some studies have focused on the comparison of caries- and health-associated microbiotas in dental plaque in order to understand the variations leading to dental demineralization [9], concluding that oral pathologies are tissue-dependent and have a polymicrobial aetiology [3,9]. In addition, several studies have shown that the frequency of certain acidogenic species appears to be related to caries status and that it can also be related to the risk of a given individual to undergo tooth decay [10].

However, the microbiota associated with one of the regions with the highest risk of caries, i.e. the interdental or interproximal (IPr) sites, has been understudied [11]. Moreover, IPr caries lesions are hard to detect and X-ray images are normally necessary, except for those cases were the dissolution of hydroxyapatite matrices of enamel and dentine is so advanced that it can be visually diagnosed [12]. Although previous studies have looked for specific pathogens associated with IPr sites, the whole microbial community has not been studied to date [13].

Previous next-generation sequencing (NGS) studies have concluded that dental plaque-associated microbiota can influence caries development [14]. Thus, a putative explanation for the higher incidence of caries in IPr sites could be a more acidogenic microbiota. In the present manuscript, we aimed to describe the IPr-associated microbial composition and to compare it with the vestibular and lingual microbiota in order to evaluate if the increased caries risk at IPr sites could be partly explained by a different bacterial community. In addition, a second sample was collected after 1 month, in order to assess the stability of those bacterial populations through time. Our data report for the first time the IPr-associated bacterial community of healthy adolescents and young adults using high-throughput Illumina sequencing of the bacterial 16S rRNA gene.

## Material and methods

### Sampling

Samples were collected from 10 patients (7 females and 3 males; 12–25 years old, Table A1) who were non-smokers, had not taken antibiotics for the last 3 months, had not used antiseptic mouthwashes in the last three months and had not performed interproximal brushing nor dental floss hygiene, had no caries lesions in premolars and had no periodontal inflammation. Air and exploration probes were used to check for caries using World Health Organization criteria while periodontal probes were used for confirming lack of periodontal inflammation. Patients were asked to toothbrush using Bass and rotational technique the night before samples were taken and during the month in between sampling days. Patients were asked to refrain from eating 1 hour before sampling and did not brush the days samples were collected. Menstrual cycles, oral consumption of contraceptives and daily times were not recorded. Samples were collected in the afternoon.

Dental plaque was sampled from the interproximal region with sterile dental floss reaching the bottom of the groove, on mesial and distal sides between upper left first and second premolars (1.4 and 1.5 teeth) and repeated again in the same patients after 1 month [13]. Vestibular (buccal) and Lingual (palatine) samples were obtained with an autoclaved spoon excavator at tooth 1.4 [5]. In both cases, the obtained plaque was placed in sterile 1.5 ml tubes with saline solution and kept at – 20ºC until DNA was extracted.

### DNA extraction

DNA extraction was performed using the MagNa Pure LC DNA Isolation kit II (Roche®) and a MagNa Pure Instrument. Protocol was used as indicated by the company with some modifications following Dzidic et al. 2018 [15]. In summary, samples were lysed using 3 × 10 seconds cycles of ultrasounds, enzymatic digestion with an enzyme cocktail of lysozyme (100 mg/ml), lysostaphin (5 kU/ml) and mutanolysin (2.5 kU/ml), followed by protein degradation with Proteinase K.

After cleaning and measuring the DNA, the V3-V4 hypervariable region of the 16S rRNA gene was amplified using universal primers optimized for Illumina sequencing, following Dzidic et al. 2018 [15]. Library was constructed following the 16S rRNA gene Metagenomic Sequencing Library Preparation Illumina protocol (Part #15,044,223 Rev. A) and sequenced at the sequencing service at the FISABIO Institute (Valencia) using the 2 × 300 bp paired-end Illumina protocol.

### Bioinformatic analyses

Reads were pre-processed before taxonomical classification. This included a length and quality filter, as well as an end-trimming procedure, following Dzidic et al. 2018 [15]. Reads were denoised and chimeras detected and eliminated. Taxonomical assignment processes were performed using dada2 [16]. The SILVA database was used as a reference to assign the reads at the genus and species level [17]. In order to assign taxonomically at the species level, a minimum of 97% of identity was established, whereas no threshold was used for genus-level assignment. If the same sequence was assigned to more than one species with the same similarity value, assignment was made at the genus level only.

Taxonomic differences among groups were assessed using Wilcoxon rank sum paired tests, with Bonferroni corrections for multiple comparisons. R was used to make heatmaps, principal component analyses (PCA) and canonical correlation analysis (CCA) to plot these differences [18].

## Results and discussion

Several studies have focused on the description of caries- or periodontitis-associated microbiota [3,8]. However, the microbiota attached to the IPr regions, where caries incidence is one of the highest and the diagnosis the hardest, has not been assessed by high-throughput sequencing [19]. Here, we sampled the IPr plaque between premolars of the first quadrant and sequenced the 16S rRNA gene in order to describe the associated microbiota. Moreover, vestibular and lingual regions were also sampled at the same time point and teeth. Finally, to assess the variability of these microbiotas over time, dental plaques were sampled again after one month. After quality filtering, we obtained an average of 71,486 ± 12,033 reads per sample. Sequences were deposited in the public repository SRA under Accession Number PRJNA545410. Microbial communities associated with the three studied regions were compared between female and male participants in order to discard a putative effect of sex. No statistically significant differences between the two groups were observed over the IPr, vestibular and lingual-associated microbiotas (CCA Analysis, Adonis p-value >0.1 in all cases). However, given the unequal distribution of age and sex, an effect of hormones on microbiota composition cannot be discarded.

### IPr-associated microbiota

**Figure 1.**
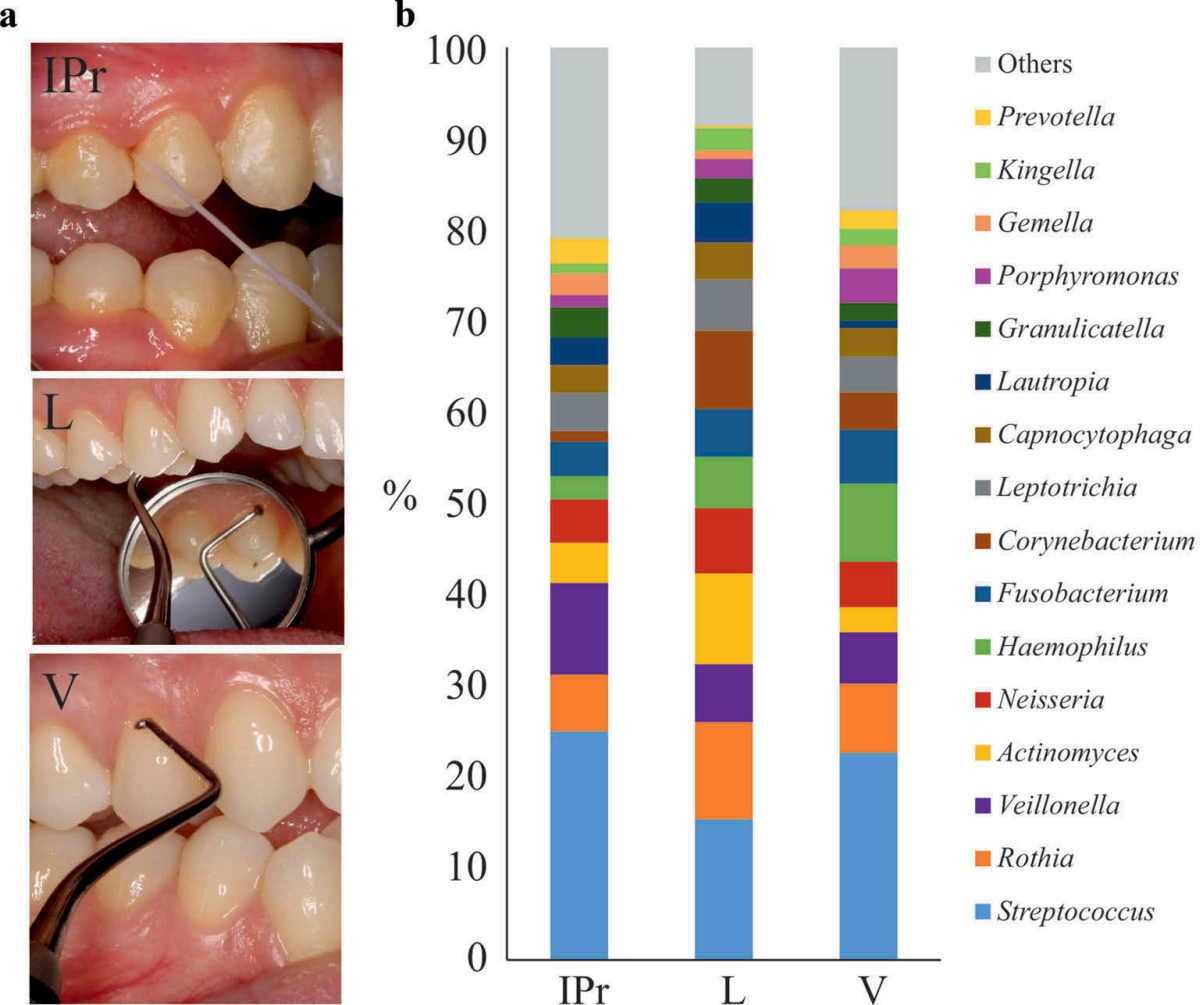
Bacterial composition at interproximal sites and their associated vestibular and lingual surfaces. (a). Dental floss sampling at the interproximal (IPr) region between teeth 1.4 and 1.5, and sampling of supragingival dental plaque at vestibular and lingual surfaces with an autoclaved spoon excavator. The pictures are merely illustrative and were taken from a different patient which was not part of the study. (b). Bacterial community composition at genus level in the three sampled regions, as determined by 16S rRNA gene Illumina sequencing

The taxonomic analysis of IPr samples showed that 80% of the microbial community was occupied by 14 genera, most of them with similar percentages. Among them, *Streptococcus* displayed the highest average value (25%) followed by *Veillonella* (10%), *Rothia* (6%) and *Neisseria* (4.7%) (Figure 1(b)).

These genera are usual members of the oral communities described so far [20]. *Rothia, Neisseria, Actinomyces, Corynebacterium* or *Haemophilus* are known commensal or symbiotic members of the oral microbiota. On the other side, *Streptococcus, Prevotella, Veillonella* and *Fusobacterium* are genera in which different species have been related to healthy and diseased conditions (caries, periodontitis, halitosis and even oral cancer) [21–25].

When the microbiota was analysed at the species level, the top 10 most abundant members were: *Streptococcus oralis* (15%), *Veillonella parvula* (6.4%), *Rothia aeria* (4.18%), *Streptococcus cristatus* (3.86%), *Granulicatella adiacens* (3.21%), *Fusobacterium nucleatum* (3%), *Scardovia wiggsiae* (2.77%), *Lautropia mirabilis* (2.62%), *Atopobium parvulum* (2.62%) and *Streptococcus sanguinis* (2.47%).

### Differences in microbiota between IPr, vestibular and lingual regions

**Figure 2.**
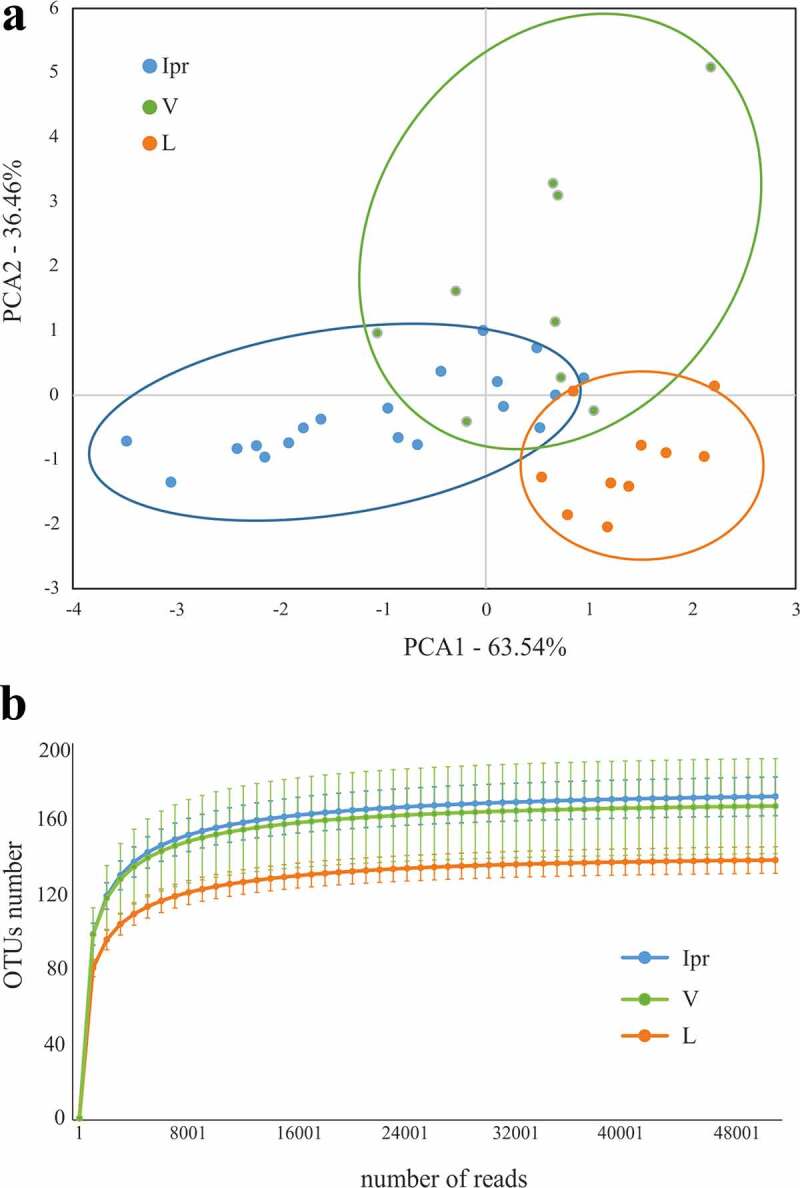
Comparison of microbiota associated with interproximal, vestibular and lingual regions. Samples are visualized in a PCA plot according to the relative abundance of genera, as determined by 16S rRNA gene Illumina sequencing (a). The estimated richness of bacterial species in each region is shown as rarefaction curves (b)

Supragingival dental plaque was also sampled from the vestibular and lingual surfaces from 1.4 teeth in the 10 patients (Figure 1(a)). Vestibular (V) and lingual–associated (L) microbiotas presented considerable similarities with IPr-associated microbial populations (Figure 1(b)). For example, *Streptococcus* was also the main genus (22.7% at V sites and 15.4% at L sites). In addition, *Veillonella* (5.6% V – 6.3% L), *Rothia* (7% V – 10% L) or *Neisseria* (5% V – 7% L) had similar percentages. Statistical analyses considering all bacteria detected, including PCA (data not shown) and CCA analyses (Figure 2(a)) showed differences among the three groups (p-value<0.005). This suggested that IPr, V and L-associated microbiotas were different and supported previous studies in which V and L microbiotas were compared [5].

**Figure 3.**
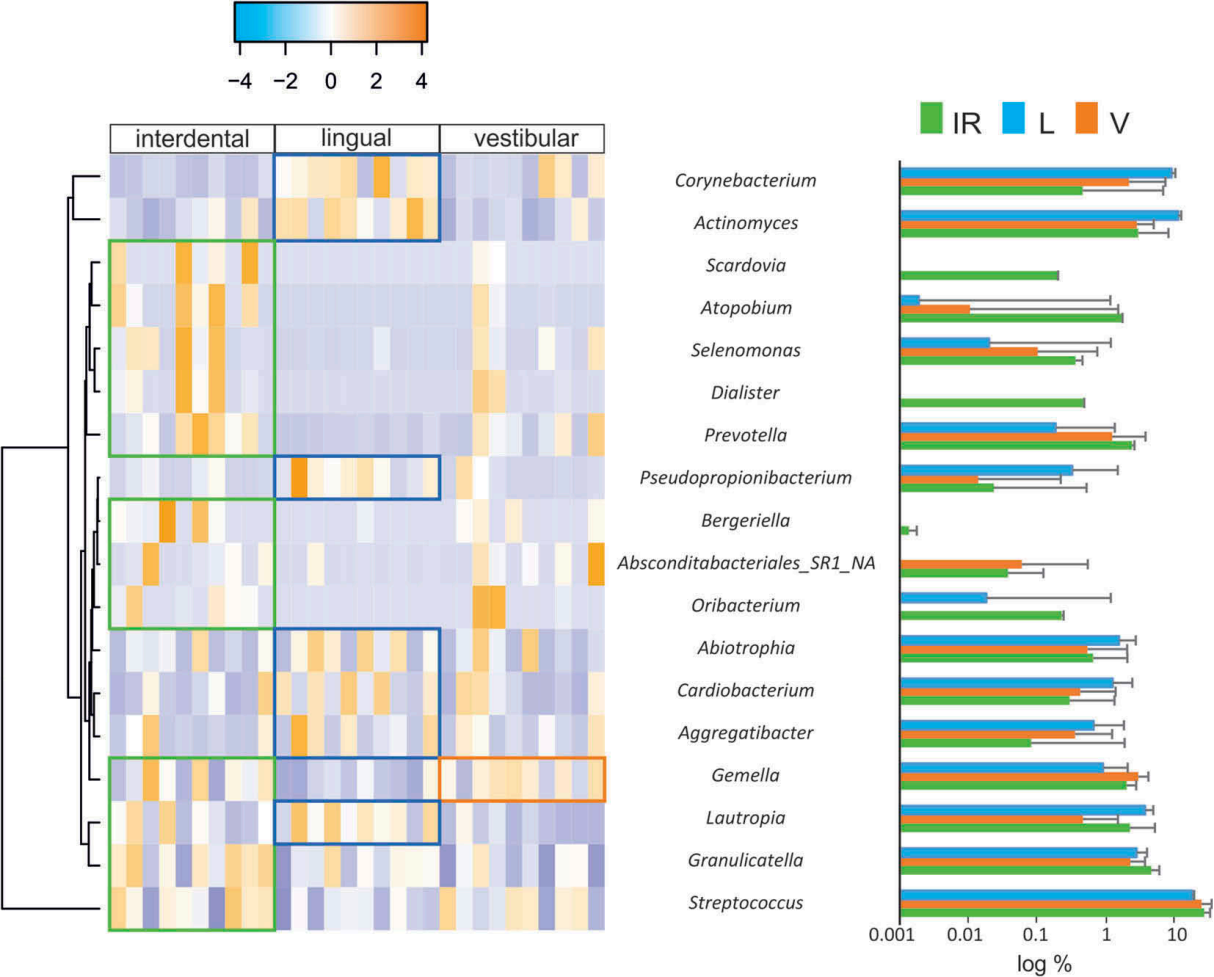
Differentially represented bacterial genera at interproximal sites and their associated vestibular and lingual regions. The abundance of differentially represented genera in the three analyzed regions is presented in a heatmap (left) and in bar plots (right). When a genus is significantly more abundant in one region, this is highlighted by box in the heatmap, and by asterisks in the bar plots (*: p-value<0.1; **: p-value<0.05)

To further identify which genera were causing the differences between groups we used Wilcox-paired tests and plotted the differences in a heatmap (Figure 3). Only two genera were differentially abundant when the IPr- and V-associated microbiotas were compared whereas the differences between IPr and L sites increased up to 16 genera. Moreover, when rarefaction curves were plotted, IPr and V bacterial communities showed higher diversity than the corresponding L samples (Figure 2(b)). This suggests that IPr microbiota was more similar to that of the equivalent vestibular regions of the teeth than to the corresponding lingual surfaces. Even though the vestibular region is more accessible to toothbrushing than IPr and therefore differences among these microbiotas would be expected, the presented results suggest that other factors could be homogenizing the microbial communities in these two regions, while the lingual bacterial community would be more unique. Other studies have also reported profound differences in bacterial composition between the lingual and vestibular surfaces, even those of the same tooth [5,26] and several environmental factors have been proposed to influence that distinct microbial composition, namely variations in pH at different locations of the mouth [27], the buffering effect of saliva, oxygen concentration [5] or access to efficient toothbrushing. In addition, the contrast in bacterial composition between the studied niches could be partly due to differences in biofilm formation. For instance, the mechanical activity of the tongue at lingual surfaces may be constantly removing the biofilm or providing colonizing inocula with tongue-associated bacteria. In addition, important bacteria for biofilm architecture, like *Fusobacterium*, were found at different levels between vestibular and lingual regions of the same teeth [5]. Thus, future work should study whether the biofilm formed at the vestibular surfaces serves as inoculum for IPr biofilm formation, or whether their similarity is a consequence of similar physico-chemical features of the two niches.

On one hand, *Corynebacterium, Actinomyces, Lautropia* and *Pseudopropionibacterium* were statistically more represented in the L region than in the V or IPr sites. Similarly, three additional genera were statistically more abundant in L sites than in the IPr region, namely *Abiotropia, Cardiobacterium* and *Aggregatibacter*. In relation to these genera, metagenomic and microscopy studies pointed at *Corynebacterium* as a key taxon in supragingival plaque architecture and composition, usually associated with health [28,29]. Similarly, *Actinomyces* has been found associated with healthy communities when compared with periodontitis [24,30]. Analysis at the species-level OTUs indicated that *Abiotrophia defectiva* was found at significantly higher levels in lingual surfaces compared to the IPr region. This species has been found in higher proportions in caries patients compared to healthy individuals [31]. However, other studies have found this bacterium associated with a caries-free microbiome [32,33]. Although *Aggregatibacter actinomycetemcomitans* has been several times associated with oral diseases [34], it was the little studied *A. aphrophilus* the species significantly more represented in the lingual region [35]. Some *Aggregatibacter* OTUs have been associated with intra-oral halitosis and periodontitis [24–26,36]. Lastly, *Cardiobacterium hominis* abundance has been correlated with aggressive periodontitis [37] and Colombo et al. found a significant reduction in this genus after treatment [38].

On the other hand, we found a group of genera at significantly higher levels in IPr than L surfaces, including *Streptococcus, Oribacterium, unknown Absconditabacteriales SR1, Bergeriella, Prevotella, Dialister, Selenomonas, Atopobium* and *Scardovia*. From these, *Prevotella* and *Dialister* are known oral members usually associated with periodontitis, caries, intra-oral halitosis and also with oral cancer [21–25], and their potential contribution to gingival inflammation, which is especially common in orthodontic patients [39–41], should be evaluated in the future. In addition, *Scardovia wiggsiae*, which has recently been shown to be strongly associated with early childhood caries, was also enriched at IPr sites in comparison with lingual surfaces [33,42]. It has to be mentioned that the abundance of *Scardovia* was highly variable among IPr samples and additional studies should confirm its enrichment in this region. Other cases in which those genera have been associated with dental diseases such as caries or periodontitis are *Atopobium* or *Selenomonas* [43,44]. This suggests that the bacteria at IPr sites may be more cariogenic than the equivalent microbial communities at the free surfaces on the lingual side from the same teeth. On the other hand, *Gemella* was found at significantly higher levels in IPr sites vs the L and V regions. This bacterium, together with *Bergeriella* and *Oribacterium*, has usually been associated with disease-free microbiotas [25,45,46].

### Variations over time

**Figure 4.**
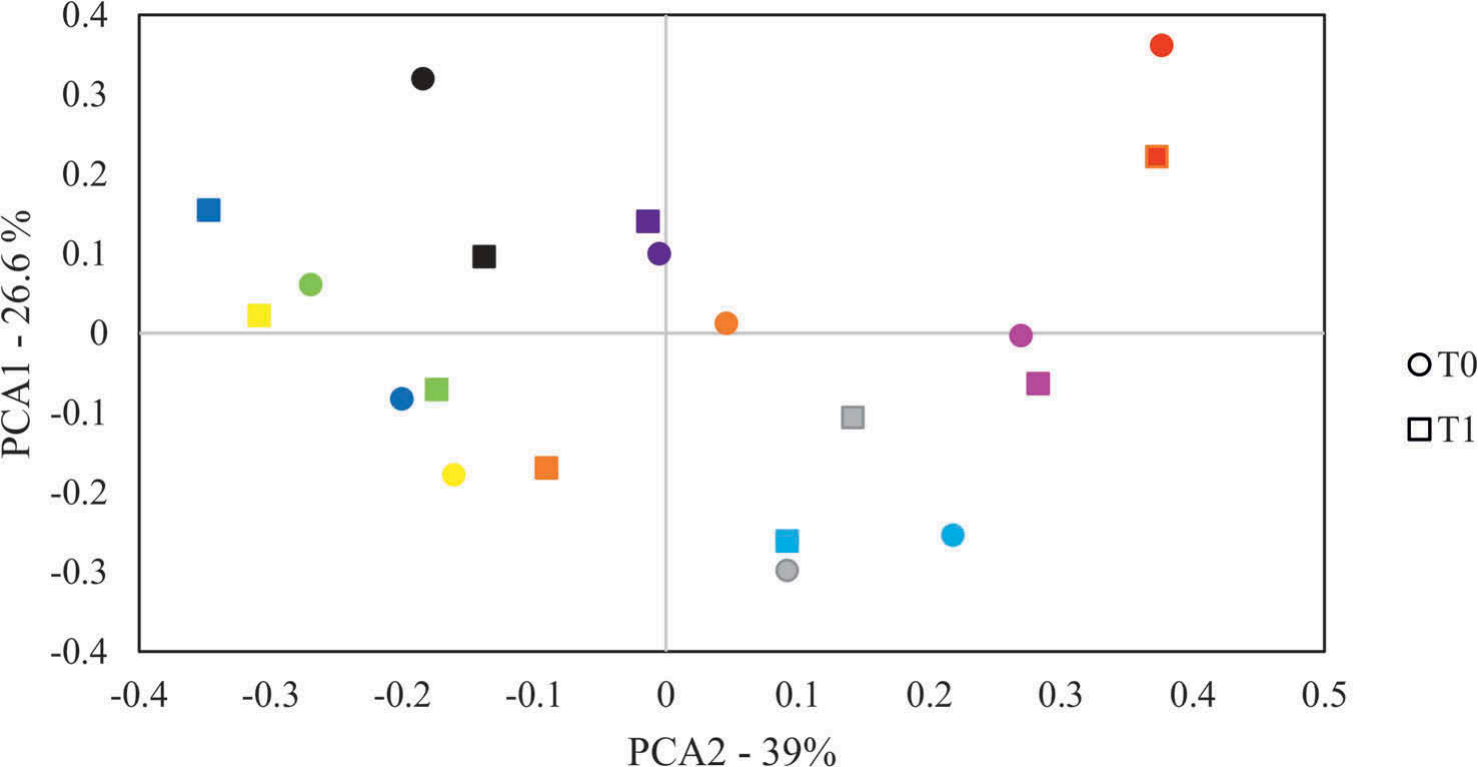
Variation of bacterial composition at the 1.4–1.5 teeth interproximal region through time. The composition at genus level was analysed by comparing the clustering of samples taken from the interproximal region at t0 and 1 month after (t1) by Principal Components Analysis (PCA). Samples from the same patient are drawn with the same colour

When comparing the IPr, V and L microbiotas at the two time points (t0 and after 1 month, t1) they appeared to be extremely similar within each patient, and only small differences were detected. Six genera were found at significantly different levels at the two time points in the IPr region, namely *Kingella, Pseudopropionibacterium, Johnsonella, Bergeriella, Capnocytophaga and Granulicatella*. However, when samples were grouped in PCA analyses we found no significant clustering by sampling time (p-value>0.1) and samples from the same individual at the two time points tended to cluster together (Figure 4). This suggested that the IPr-associated microbiota remain stable after one month in the healthy patients studied and supports that although the amount of interproximal plaque that can be collected with dental floss is limited, this sampling method appears to be reproducible. It also suggests that one month after sampling, the IPr biofilm is fully restored, and that the bacterial communities inhabiting the interdental niche are mainly unaffected by standard toothbrushing. Similarly, V- and L-associated microbiotas were stable over time and only one genus presented variations at the two time points, namely *Rothia* and *Johnsonella*, respectively. It is difficult to know why these genera changed in proportion between the two time points, and it could be part of the normal variability in oral microbiota composition, which has been shown to have certain degree of fluctuation at different time points [47]. Thus, the data suggest that vestibular, lingual and IPr sites are microbiologically distinct niches that contain a relatively stable bacterial composition through time.

## Conclusions

In conclusion, the presented results suggested that the different environmental conditions at the IPr region may influence the associated microbiota, which appears to be specific of this niche and stable through time. Although the differences of the microbial composition between IPr sites and vestibular and lingual regions are relatively small, some genera that appear to be more represented in the IPr areas than in lingual surfaces (such as *Prevotella, Dialister, Scardovia, Atopobium* or *Selenomonas*) have been associated with oral diseases. Therefore, we hypothesize that if the appropriate conditions for caries development are present, the IPr-associated microbiota may increase the risk for caries development and gingival inflammation, at least in comparison with the lingual region. This could be particularly relevant after orthodontic treatment, and future work should study the potential changes in IPr microbiota induced by brackets or other orthodontic appliances.

## Appendix

**Table A1.** Patients’ dataset

Patient	Sex	Age
1	F	16
2	F	25
3	M	13
4	F	17
5	F	12
6	F	15
7	M	13
8	F	15
9	M	19
10	F	12–13

F, female M, male

## References

[1] Arweiler NB, Netuschil L. The oral microbiota. Adv Exp Med Biol. 2016;902:45–8. 27161350 10.1007/978-3-319-31248-4_4

[2] Gao L, Xu T, Huang G, et al. Oral microbiomes: more and more importance in oral cavity and whole body. Protein Cell. 2018 5;9(5):488–500. 29736705 10.1007/s13238-018-0548-1 PMC5960472

[3] Simón-Soro Á, Belda-Ferre P, Cabrera-Rubio R, et al. A Tissue-dependent hypothesis of dental caries. Caries Res. 2013;47(6):591–600. 24080530 10.1159/000351663

[4] Segata N, Haake SK, Mannon P, et al. Composition of the adult digestive tract bacterial microbiome based on seven mouth surfaces, tonsils, throat and stool samples. Genome Biol. 2012 6 14;13(6):R42. 22698087 10.1186/gb-2012-13-6-r42 PMC3446314

[5] Simón-Soro A, Tomás I, Cabrera-Rubio R, et al. Microbial geography of the oral cavity. J Dent Res. 2013 7;92(7):616–621. 23674263 10.1177/0022034513488119

[6] Nyvad B, Crielaard W, Mira A, et al. Dental caries from a molecular microbiological perspective. Caries Res. 2013;47(2):89–102. 23207320 10.1159/000345367

[7] Hajishengallis G, Darveau RP, Curtis MA. The keystone-pathogen hypothesis. Nat Rev Microbiol. 2012 10 3;10(10):717–725. 22941505 10.1038/nrmicro2873 PMC3498498

[8] Mira A, Simon-Soro A, Curtis MA. Role of microbial communities in the pathogenesis of periodontal diseases and caries. J Clin Periodontol. 2017 3;44(Suppl 1):S23–38. 28266108 10.1111/jcpe.12671

[9] Belda-Ferre P, Alcaraz LD, Cabrera-Rubio R, et al. The oral metagenome in health and disease. Isme J. 2012 1;6(1):46–56. 21716308 10.1038/ismej.2011.85 PMC3246241

[10] Hemadi AS, Huang R, Zhou Y, et al. Salivary proteins and microbiota as biomarkers for early childhood caries risk assessment. Int J Oral Sci. 2017;9(11):e1. 29125139 10.1038/ijos.2017.35 PMC5775330

[11] Cagetti MG, Campus G, Sale S, et al. Association between interdental plaque acidogenicity and caries risk at surface level: a cross sectional study in primary dentition. Int J Paediatr Dent. 2011 3;21(2):119–125. 20731733 10.1111/j.1365-263X.2010.01099.x

[12] Senel B, Kamburoglu K, Uçok O, et al. Diagnostic accuracy of different imaging modalities in detection of proximal caries. Dentomaxillofac Radiol. 2010 12;39(8):501–511. 21062944 10.1259/dmfr/28628723 PMC3520212

[13] Bourgeois D, David A, Inquimbert C, et al. Quantification of carious pathogens in the interdental microbiota of young caries-free adults. Hozbor DF, editor. PLoS One. 2017 10 10;12(10):e0185804. 29016613 10.1371/journal.pone.0185804 PMC5634565

[14] Simón-Soro A, Mira A. Solving the etiology of dental caries. Trends Microbiol. 2015 2;23(2):76–82. 25435135 10.1016/j.tim.2014.10.010

[15] Dzidic M, Collado MC, Abrahamsson T, et al. Oral microbiome development during childhood: an ecological succession influenced by postnatal factors and associated with tooth decay. Isme J. 2018 9;12(9):2292–2306. 29899505 10.1038/s41396-018-0204-z PMC6092374

[16] Callahan BJ, McMurdie PJ, Rosen MJ, et al. DADA2: high-resolution sample inference from Illumina amplicon data. Nat Methods. 2016;13(7):581–583. 27214047 10.1038/nmeth.3869 PMC4927377

[17] Quast C, Pruesse E, Yilmaz P, et al. The SILVA ribosomal RNA gene database project: improved data processing and web-based tools. Nucleic Acids Res. 2013 1;41(Database issue):D590–6. 23193283 10.1093/nar/gks1219 PMC3531112

[18] R Development Core Team. R: A language and environment for statistical computing. R Foundation for Statistical Computing, Vienna, Austria. 2016.

[19] Chestnutt IG, Schafer F, Jacobson AP, et al. Incremental susceptibility of individual tooth surfaces to dental caries in Scottish adolescents. Community Dent Oral Epidemiol. 1996 2;24(1):11–16. 8833507 10.1111/j.1600-0528.1996.tb00804.x

[20] Dewhirst FE, Chen T, Izard J, et al. The human oral microbiome. J Bacteriol. 2010 10 1;192(19):5002–5017. 20656903 10.1128/JB.00542-10 PMC2944498

[21] Gonçalves C, Soares GMS, Faveri M, et al. Association of three putative periodontal pathogens with chronic periodontitis in Brazilian subjects. J Appl Oral Sci. 2016 4;24(2):181–185. 27119767 10.1590/1678-775720150445 PMC4836926

[22] Zhao H, Chu M, Huang Z, et al. Variations in oral microbiota associated with oral cancer. Sci Rep. 2017 12 18;7(1):11773. 28924229 10.1038/s41598-017-11779-9 PMC5603520

[23] Wang Y, Zhang J, Chen X, et al. Profiling of oral microbiota in early childhood caries using single-molecule real-time sequencing. Front Microbiol. 2017;8:2244. 29187843 10.3389/fmicb.2017.02244 PMC5694851

[24] Chen C, Hemme C, Beleno J, et al. Oral microbiota of periodontal health and disease and their changes after nonsurgical periodontal therapy. Isme J. 2018 5 16;12(5):1210–1224. 29339824 10.1038/s41396-017-0037-1 PMC5932080

[25] Seerangaiyan K, van Winkelhoff AJ, Harmsen HJM, et al. The tongue microbiome in healthy subjects and patients with intra-oral halitosis. J Breath Res. 2017 9 6;11(3):036010. 28875948 10.1088/1752-7163/aa7c24

[26] Haffajee AD, Teles RP, Patel MR, et al. Factors affecting human supragingival biofilm composition. II. Tooth position. J Periodontal Res. 2009 8;44(4):520–528. 18973539 10.1111/j.1600-0765.2008.01155.x PMC2710397

[27] Kleinberg I, Jenkins GN. The pH of dental plaques in the different areas of the mouth before and after meals and their relationship to the pH and rate of flow of resting saliva. Arch Oral Biol. 1964;9(5):493–516. 14205453 10.1016/0003-9969(64)90015-9

[28] Eriksson L, Lif Holgerson P, Johansson I. Saliva and tooth biofilm bacterial microbiota in adolescents in a low caries community. Sci Rep. 2017;7(1):5861. 28724921 10.1038/s41598-017-06221-z PMC5517611

[29] Mark Welch JL, Rossetti BJ, Rieken CW, et al. Biogeography of a human oral microbiome at the micron scale. Proc Natl Acad Sci U S A. 2016 2 9;113(6):E791–800. 26811460 10.1073/pnas.1522149113 PMC4760785

[30] Liu B, Faller LL, Klitgord N, et al. Deep sequencing of the oral microbiome reveals signatures of periodontal disease. PLoS One. 2012;7(6):e37919. 22675498 10.1371/journal.pone.0037919 PMC3366996

[31] ElSalhy M, Söderling E, Honkala E, et al. Salivary microbiota and caries occurrence in Mutans Streptococci-positive school children. Eur J Paediatr Dent. 2016 9;17(3):188–192. 27759406

[32] Kanasi E, Dewhirst FE, Chalmers NI, et al. Clonal analysis of the microbiota of severe early childhood caries. Caries Res. 2010;44(5):485–497. 20861633 10.1159/000320158 PMC2975730

[33] Jiang W, Ling Z, Lin X, et al. Pyrosequencing analysis of oral microbiota shifting in various caries states in childhood. Microb Ecol. 2014 5 7;67(4):962–969. 24504329 10.1007/s00248-014-0372-y

[34] Fine DH, Patil AG, Velusamy SK. *Aggregatibacter actinomycetemcomitans* (Aa) Under the radar: myths and misunderstandings of Aa and its role in aggressive periodontitis. Front Immunol. 2019;10:728. 31040843 10.3389/fimmu.2019.00728 PMC6476972

[35] Lucchese A, Bondemark L, Marcolina M, et al. Changes in oral microbiota due to orthodontic appliances: a systematic review. J Oral Microbiol. 2018;10(1):1476645. 29988826 10.1080/20002297.2018.1476645 PMC6032020

[36] Chen W-P, Chang S-H, Tang C-Y, et al. Composition analysis and feature selection of the oral microbiota associated with periodontal disease. Biomed Res Int. 2018;2018:3130607. 30581850 10.1155/2018/3130607 PMC6276491

[37] Lourenço TGB, Heller D, Silva-Boghossian CM, et al. Microbial signature profiles of periodontally healthy and diseased patients. J Clin Periodontol. 2014 11;41(11):1027–1036. 25139407 10.1111/jcpe.12302 PMC4213353

[38] Colombo APV, Bennet S, Cotton SL, et al. Impact of periodontal therapy on the subgingival microbiota of severe periodontitis: comparison between good responders and individuals with refractory periodontitis using the human oral microbe identification microarray. J Periodontol. 2012 10;83(10):1279–1287. 22324467 10.1902/jop.2012.110566 PMC3971922

[39] Ren Y, Jongsma MA, Mei L, et al. Orthodontic treatment with fixed appliances and biofilm formation–a potential public health threat? Clin Oral Investig. 2014 9;18(7):1711–1718. 10.1007/s00784-014-1240-3 24728529

[40] Goes P, Dutra CS, Lisboa MRP, et al. Clinical efficacy of a 1% Matricaria chamomile L. mouthwash and 0.12% chlorhexidine for gingivitis control in patients undergoing orthodontic treatment with fixed appliances. J Oral Sci. 2016;58(4):569–574. 28025442 10.2334/josnusd.16-0280

[41] Jiang Q, Li J, Mei L, et al. Periodontal health during orthodontic treatment with clear aligners and fixed appliances. J Am Dent Assoc. 2018 8;149(8):712–720.e12. 29921415 10.1016/j.adaj.2018.04.010

[42] Kressirer CA, Smith DJ, King WF, et al. Scardovia wiggsiae and its potential role as a caries pathogen. J Oral Biosci. 2017 8;59(3):135–141. 29104444 10.1016/j.job.2017.05.002 PMC5665406

[43] Aas JA, Griffen AL, Dardis SR, et al. Bacteria of dental caries in primary and permanent teeth in children and young adults. J Clin Microbiol. 2008 4;46(4):1407–1417. 18216213 10.1128/JCM.01410-07 PMC2292933

[44] Kianoush N, Adler CJ, Nguyen K-AT, et al. Bacterial profile of dentine caries and the impact of pH on bacterial population diversity. Burne RA, editor. PLoS One. 2014 3 27;9(3):e92940. 24675997 10.1371/journal.pone.0092940 PMC3968045

[45] He J, Tu Q, Ge Y, et al. Taxonomic and functional analyses of the supragingival microbiome from caries-affected and caries-free hosts. Microb Ecol. 2018 2;75(2):543–554. 28932895 10.1007/s00248-017-1056-1

[46] Tsai C-Y, Tang CY, Tan T-S, et al. Subgingival microbiota in individuals with severe chronic periodontitis. J Microbiol Immunol Infect. 2018 4;51(2):226–234. 27262209 10.1016/j.jmii.2016.04.007

[47] Caporaso JG, Lauber CL, Costello EK, et al. Moving pictures of the human microbiome. Genome Biol. 2011;12(5):R50. 21624126 10.1186/gb-2011-12-5-r50 PMC3271711

